# Decompression Retinopathy after ExPRESS Shunt Implantation for Steroid-Induced Ocular Hypertension: A Case Report

**DOI:** 10.1155/2011/303287

**Published:** 2011-12-21

**Authors:** Khawla Abu Samra, Sandra Fernando Sieminski, Vimal Sarup

**Affiliations:** Ross Eye Institute, The State University of New York at Buffalo, Buffalo, NY 14209, USA

## Abstract

*Purpose*. To present a unique case of decompression retinopathy after the implantation of ExPRESS drainage device. *Method*. A 25-year-old female patient underwent implantation of ExPRESS drainage device in the left eye for the management of steroid-induced ocular hypertension. *Results*. On the postoperative day one, best-corrected visual acuity in the left eye was 20/50. Fundus examination revealed diffuse intraretinal hemorrhages, some white-centered, throughout the retina. There was also marked tortuosity to the retinal vasculature and no evidence of choroidal effusion. Intravenous fluorescein angiography and indocyanine green did not contribute to the aetiopathogenesis. *Conclusion*. Decompression retinopathy can occur following the implantation of ExPRESS drainage device. It is very important to be aware of this complication in patients with relatively high intraocular pressure who is planned for filtration surgery, including the ExPRESS implant.

## 1. Introduction

Decompression retinopathy, characterized by the transient appearance of scattered retinal hemorrhages in the immediate postoperative period following glaucoma surgery, was first described in 1992 by Fechtner et al. as a complication of trabeculectomy [1]. The syndrome has been also recognized in relation to other glaucoma penetrating procedures such as Ahmed valve implantation and deep sclerotomy as well as nonpenetrating procedures such as peripheral iridotomy [2–6]. Herein, we present a unique case of decompression retinopathy following the implantation of an ExPRESS drainage device (Alcon Laboratories, Fort Worth, TX, USA) in a young patient with steroid-induced ocular hypertension following penetrating keratoplasty for keratoconus.

## 2. Case Report

A 25-year-old female patient, a known asthmatic on albuterol, underwent penetrating keratoplasty for keratoconus in the left eye. Due to early graft rejection, she received a postoperative subtenons kenalog injection and subsequently developed steroid-induced ocular hypertension. Maximum intraocular pressure (IOP) after subtenon kenalog injection was 44 mm Hg and best-corrected visual acuity was 20/20 in the right eye and 20/25 in the left eye. The remainder of the slit lamp examination revealed Vogt's striae in the right eye and clear lenses in both eyes. Fundus examination showed healthy optic nerves bilaterally with a vertical cup-to-disc ratio of 0.4 in both eyes. Humphrey visual field testing (24–2) was full in both eyes.

The decision was made to perform ExPRESS drainage device implantation in the left eye (Alcon Laboratories, Fort Worth, TX, USA) due to sustained elevated intraocular pressures despite maximum medical therapy. Acetazolamide was discontinued one day preoperatively, and all glaucoma medications were discontinued on the day of surgery. A fornix-based conjunctival dissection was created, after a subtenon injection of 1% preservative-free lidocaine was injected superiorly. A 3 mm × 3 mm partial thickness rectangular flap was created, and sponges soaked in 0.4 mg/mL mitomycin C were administered to the area for 2 minutes. The sponges were removed, and the area was irrigated with balanced salt solution. The anterior chamber was entered with a 26-gauge needle under the scleral flap in the center of the blue-gray transition zone, and an ExPRESS drainage device, model P-50, was introduced into the anterior chamber. The scleral flap was subsequently closed with 3 interrupted 10–0 nylon flap sutures, and the conjunctiva and Tenon's fascia were reapproximated to the limbus with four 10–0 nylon wing sutures. Viscoelastic was not injected into the anterior chamber prior to ExPRESS implantation; however, the anterior chamber was maintained throughout the procedure. 

**Figure 1:**
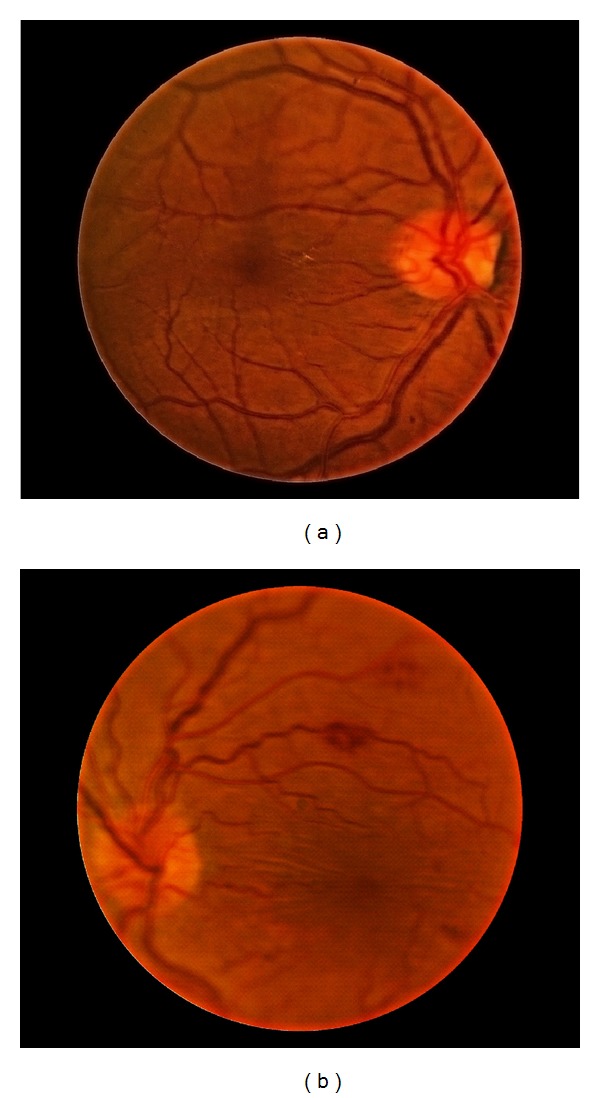
(a) Right eye: normal. (b) Left eye: diffuse intraretinal hemorrhages, some white-centered, with macular striae.

**Figure 2:**
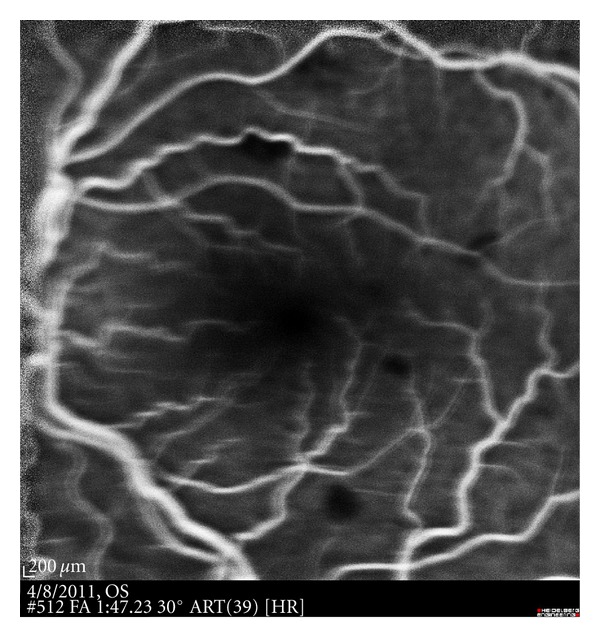
Late phase of a fluorescein angiogram demonstrates blocking defects due to overlying retinal haemorrhages.

On postoperative day one, best-corrected visual acuity in the left eye was 20/50, and the IOP was 4 mm Hg, with a diffuse nonleaking superior bleb and a well-formed anterior chamber. The corneal graft was clear. Fundus examination of left eye revealed diffuse intraretinal hemorrhages, some white-centered, throughout the retina. There was also marked tortuosity to the retinal vasculature and no evidence of choroidal effusion. The fundus examination of the right eye was unremarkable. The patient was started on prednisolone acetate 1% every 2 hrs, atropine 1% twice daily, and oral prednisone 40 mg per day. One week later, visual acuity was maintained, and IOP was 3 mm Hg with diffuse nonleaking bleb and deep anterior chamber. Fundus examination showed resolving retinal hemorrhages with macular striae (Figure 1). Intravenous fluorescein angiography and indocyanine green did not contribute to the aetiopathogenesis (Figure 2).

One month later, the visual acuity in the left eye improved to baseline of 20/25 with a diffuse nonleaking bleb, and the IOP was 7 mm Hg. The fundus showed partial resolution of all hemorrhages and macular striae. Three months postoperatively, the IOP was 12 mm Hg with complete resolution of all retinal changes.

## 3. Discussion

Decompression retinopathy is a rare complication that typically appears after glaucoma surgery [1]. The usual manifestations include transient scattered retinal hemorrhages that involve the posterior pole, which resolve within a few weeks, and an unaffected or slightly reduced visual acuity [1, 7]. Although originally described in association with trabeculectomy [1], decompression retinopathy has been reported in a variety of penetrating and nonpenetrating glaucoma procedures [2–6, 8].

Our patient developed diffuse unilateral retinopathy after the implantation of ExPRESS implant without prior history of retinopathy. The diagnosis of retinal decompression was based on a typical history, retinal findings, and the exclusion of other possible causes, namely, valsalva retinopathy and retinal vein occlusion. As our patient had retinopathy only in the operated eye, it was unlikely to be a case of valsalva retinopathy, which is a bilateral condition. In addition, our patient was not operated under general anesthesia that is often associated with increased intrathoracic pressure at time of extubation. Our patient is young, with no history of coagulopathy, making retinal vein occlusion unlikely. Additionally, fluorescein angiography aided in the exclusion of this diagnosis.

The ExPRESS drainage device is a nonvalved stainless steel device that has been shown to be a safe procedure when implanted under a scleral flap [9, 10]. In comparison to traditional trabeculectomy, ExPRESS implantation has the advantage of decreased early postoperative complications and faster visual recovery [11, 12]. Some have concluded that ExPRESS implantation is most appropriate in patients in whom the risk of complications, such as hypotony, is high [13, 14]. The case described in this paper is, to the best of our knowledge, unique in the literature and has not been reported previously in relation to ExPRESS implantation.

There are several hypotheses in the pathogenesis of decompression retinopathy [1, 15]. The most widely described hypothesis is that of impaired autoregulation of the retinal vasculature, in which acutely lowering the IOP increases blood flow through the retinal capillary bed leading to multiple focal leaks presenting as blot hemorrhages [1]. This process is more likely in patients whose autoregulation capacity of the retinal vasculature is impaired, such as patients with longstanding glaucoma. Fechtner et al. also proposed that with acute IOP lowering, forward movement of the lamina cribrosa occurs, causing an acute blockage of axonal transport and subsequent decompression retinopathy [1]. Lastly, Kozobolis et al. proposed that retinal toxicity with intraoperative mitomycin C use may potentiate decompression retinopathy [3].

In our patient, the history of a high preoperative intraocular pressure developing over a short period of time may have been a predisposing factor to defective autoregulation. The sudden reduction in IOP following the implantation of the ExPRESS drainage device presumably resulted in a large increase in the retinal arterial perfusion pressure. We hypothesize that this increase overwhelmed the autoregulatory capacity of the retinal vasculature, resulting in multiple hemorrhages. Other causes of increased capillary fragility, such as inherited or acquired vascular disorders [16] or medications such as aspirin which could also explain the acute occurrence of retinal hemorrhages, were not risk factors associated with our patient. Additionally, acute reduction in IOP may result in structural changes to the eye leading to scleral collapse [16]. These structural changes may, in turn, lead to capillary fragility and subsequent retinal hemorrhages [15]. No such changes of the globe occurred during the surgery in our patient.

We propose that patients with persistently high IOP, particularly developing over a short period of time, are at an increased risk of decompression retinopathy following filtration surgery. This case demonstrates that decompression retinopathy can occur following the implantation of ExPRESS drainage device. It is advisable to prepare for and counsel patients regarding this complication when the implantation of such device is planned.
